# The incidence of severe intraventricular hemorrhage based on retrospective analysis of 35939 full-term newborns—report of two cases and review of literature

**DOI:** 10.1007/s00381-016-3164-5

**Published:** 2016-07-08

**Authors:** Dawid Szpecht, Dagmara Frydryszak, Norbert Miszczyk, Marta Szymankiewicz, Janusz Gadzinowski

**Affiliations:** 1 Department of Neonatology, Poznan University of Medical Sciences in Poznań, Polna 33 Street, Poznań, Poland; 2 Student Scientific Group of Perinatal Medicine, Poznan University of Medical Sciences, Poznań, Poland

**Keywords:** Intraventricular hemorrhage, Term neonate, Etiopathogenesis

## Abstract

**Introduction:**

Intraventricular hemorrhage (IVH) is mostly documented in premature infants, and the younger the gestational age, the more often it occurs. IVH is very rarely reported in full-term neonates.

**Case report:**

Retrospective analysis was performed in 35939 full-term neonates, who were born in the Clinical Hospital of Gynecology and Obstetrics at the University of Medical Sciences in Poznań. Clinical data were retrieved from their medical records. We report a case series of 2 term newborns, who developed severe IVH grade 3 and 4 with no evidence of asphyxia, neuroinfection, TORCH infections, coagulation disorders and trombocytopenia, metabolic disorders, arteriovenous malformations, and selected genetic abnormalities (factor V Leiden 1601G > A polymorphism and MTHFR 677C > T; 1298A > C polymorphisms). IVH in both cases was complicated by posthemorrhagic hydrocephalus treated with decompressive lumbar punctures and next ventriculoperitoneal shunt placement.

**Conclusions:**

In conclusion, several factors influence the predisposition for severe IVH in term neonates. Perinatal period complicated by fetal distress, birth trauma, and severe asphyxia should be taken into account. However, it is possible that etiopathogenesis cannot be defined clearly as in our cases. Cranial ultrasounds in a specific group of term newborns (taking into account risk factors for IVH) should be widely recommended.

## Introduction

Intraventricular hemorrhage (IVH) is mostly documented in premature infants, and the younger the gestational age, the more often it occurs [1]. IVH is very rarely reported in full-term neonates and may occur in these children with a variety of clinical conditions, mostly due to perinatal trauma, asphyxia, and coagulation disorders. Very often, IVH in full-term neonates comes from choroidal plexus and is connected with venous thrombosis and ischemia of the thalamus. However, in some cases, it might be a result of damage to the residual periventricular germinal matrix. The high mortality rate in full-term newborns with IVH is explained by perinatal asphyxia [2].

Retrospective analysis of 35939 full-term neonates was performed. Children were born from January 1, 2009 to December 31, 2014 in the Clinical Hospital of Gynecology and Obstetrics at Poznan University of Medical Sciences. Cranial ultrasounds were performed according to local standards. We reported two full-term babies suffering from grade 3 and 4 of IVH. The incidence rate of IVH grade 3 and 4 was 5.5 per 100,000 live term births.

### Case 1

A 39-week gestation of male neonate weighing 3670 g was born in good general condition by vaginal delivery to a 39-year-old gravida 4, para 4 mother. The pregnancy was complicated by maternal diabetes. Apgar scores were 9 at first minute and then 9 and 10 at third and fifth minutes, respectively. Umbilical cord pH was measured at 7.13 (BE −9.8 mEq/l) and 7.14 (BE −9.3 mEq/l). The amniotic fluid was meconium-stained.

After delivery, the newborn presented tachypnea, radiological signs of pneumonia, and increased inflammatory markers (C-reactive protein concentration was 16.39 mg/l and white blood cell count was 22.66 G/l). Further investigation showed *Proteus mirabilis* from a throat swab and navel culture. The patient was treated with broad-spectrum antibiotics for 7 days. According to the results of laboratory tests, the neuroinfection, TORCH (toxoplasmosis–other–rubella–cytomegalovirus–herpesviridae) infections, coagulation disorders and trombocytopenia, metabolic disorders, and selected genetic abnormalities (factor V Leiden 1601G > A polymorphism and MTHFR 677C > T; 1298A > C polymorphisms) were excluded.

Birth tremors of the upper and lower limbs as well as inconsolable cry were observed. On the third day of life, a single episode of focal seizures of the left upper limb, increased muscle tone in limbs (especially on the left side), excessive tendon reflexes, and neck stiffness were presented. The first cranial ultrasound was performed on the third day of life and revealed extension of the both right and left lateral ventricles with features of third stage IVH. In occipital areas, there were hemorrhagic foci (fourth stage IVH according to papilla criteria). In subsequent ultrasounds, we confirmed posthemorrhagic hydrocephalus.

**Fig. 1:**
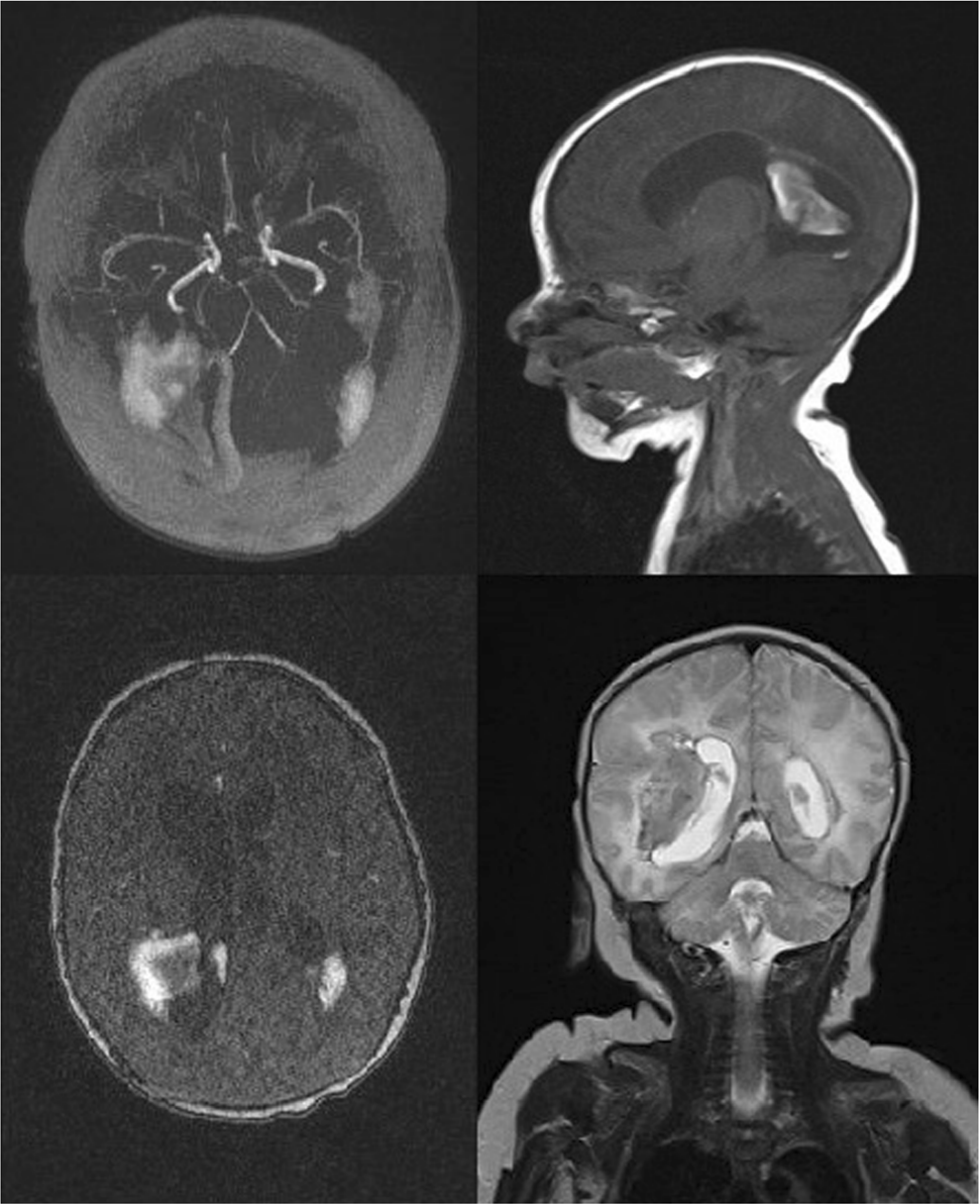
Brain magnetic resonance imaging of the neonate reported as case 1. The right hemisphere of the brain on the border of the temporoparietal lobes, an intracerebral hematoma, second in the left temporal lobe

Magnetic resonance imaging (MRI) showed in the right hemisphere of the brain on the border of the temporoparietal lobes, an intracerebral hematoma—transverse dimensions approximately 3 × 2 cm. A second, sized 2 × 1 cm was visible in the left temporal lobe, at the lower left corner of the lateral ventricle. The ventricular system was not displaced, supratentorially dilated with significant asymmetric dilation of lateral ventricles. Angio-MR showed the main intracranial arterial trunks. There was no evidence of significant vascular defect (Fig. 1).

### Case 2

A male full-term neonate weighing 3840 g was born in good general condition, by vaginal delivery to a first-time mother at 39 weeks of gestation. The pregnancy was without any complications. Apgar scores were 9, 9 and 10 at first, third, and fifth minutes, respectively. Umbilical cord pH was measured at 7.06 (BE −12.1 mEq/l) and 7.14 (BE −10.5 mEq/l).

On the third day of life screening, cranial ultrasound (in accordance with local recommendations in patients with pH below 7.1) was performed. According to papilla criteria, IVH stage 3 to the left lateral ventricle and stage 2 to the right lateral ventricle were diagnosed. In subsequent ultrasounds, performed every 3 days, we confirmed posthemorrhagic hydrocephalus.

Laboratory tests and microbiological research excluded neuro and intrauterine infection. Coagulation disorders and thrombocytopenia, TORCH infections, selected genetic abnormalities (factor V Leiden (F5) 1601G > A polymorphism and MTHFR 677C > T; 1298A > C polymorphisms), and metabolic disorders were also excluded.

In neurologic assessment, a decreased muscle tone along the head-torso axis and an increased tone in the upper and lower limbs were detected. Physiotherapy treatment (Vojta method) and stimulation of the sucking reflex were applied during the time of hospitalization.

**Fig. 2:**
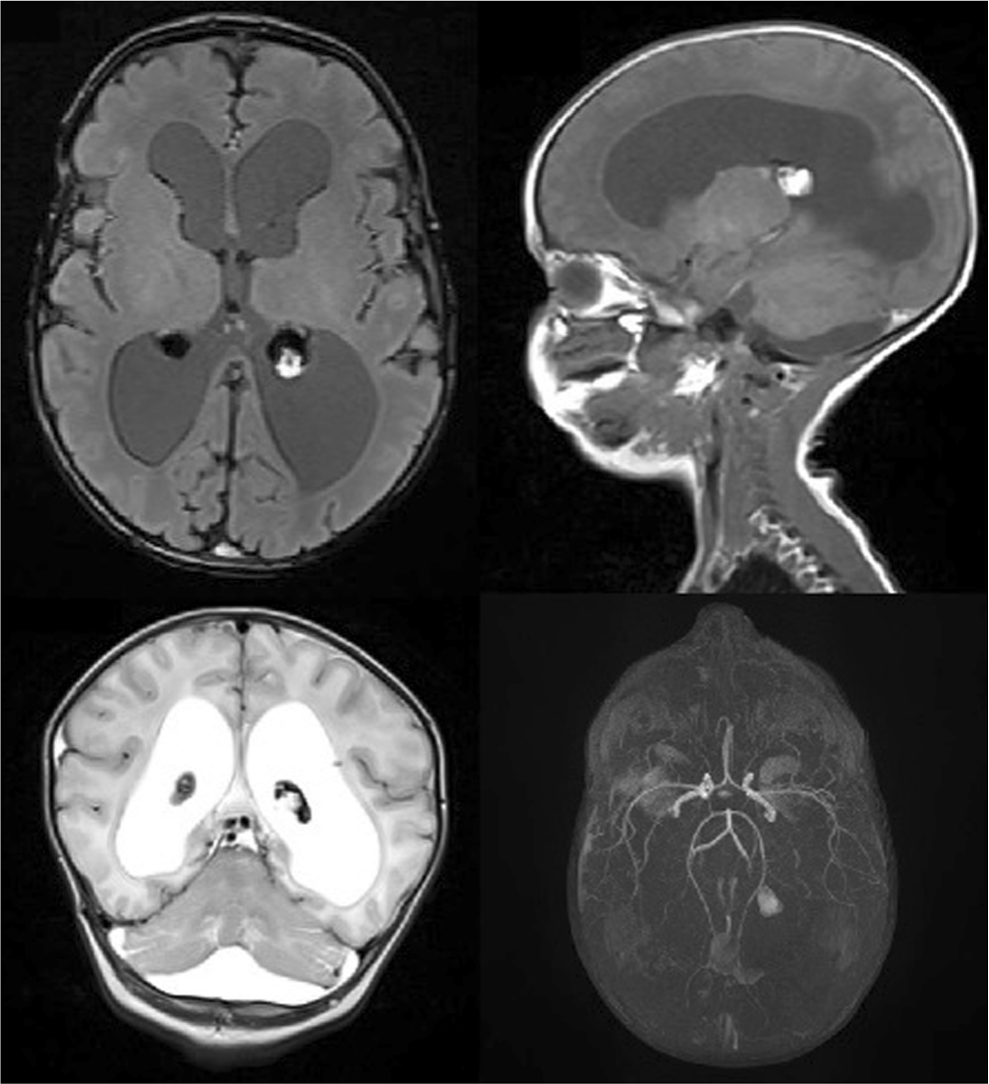
Brain magnetic resonance imaging of the neonate reported as case 2. Hydrocephalus and posthemorrhagic changes in both choroid plexuses, in the left choroid plexus fresh bleeding focus

To verify the diagnosis and identify etiopathogenesis of IVH on the 22nd day of life, the patient underwent MRI. The MRI showed asymmetric, dilated ventricular system and in both choroidal plexuses visualized posthemorrhagic changes and in left choroidal plexus fresh bleeding focus. In midline, straight under tentorium cerebella, there were signs of fresh bleeding. Angio-MR did not show any vascular defects and obstructions within the major intracranial arteries (Fig. 2).

Both patients were discharged to the Department of Pediatric Neurosurgery for further treatment (ventriculoperitoneal shunt placement). Patient reported as case 1 developed cerebral palsy at age 18 months (diplegia spastica) with non-verbal communications skills impairment. In second child, at age 18 months, mild gross motor (walk on knee and able to stand and walk with support) and fine motor delays without any non-verbal communications skills impairment or vision abnormalities were observed and required physical therapy.

## Discussion

### Incidence and clinical features

IVH is an important source of neonatal morbidity and mortality. The incidence of IVH in term newborns is not known. The incidence of all types of symptomatic intracranial hemorrhages (epidural, subdural, subarachnoid, intraventricular, and intraparenchymal) is 0.27–0.49 per 1000 live births [1]. IVH in full-term infants usually occurs during labor due to mechanical factors; however, in the preterm infants, it occurs mostly as a result of the immature central nervous system and hemodynamic instability. Besides etiology, the location of hemorrhage, clinical presentation, and neurological outcome also differs in the term and preterm infants. The term newborn with IVH typically presents with signs such as seizures, apnea, irritability or lethargy, and vomiting with dehydration. Flaccidity, loss of pupillary reaction, extraocular movements, coma, irritability, vomiting, shrill cry, central facial weakness, opisthotonic posturing, fever or hypothermia, hypo- or hyperglycemia, decreased lower extremity tone, neck flexor hypotonia, head lag, and brisk reflexes are less frequent in term newborns, however more often observed in preterms. In both cases described by us, seizures and increased muscle tone in the limbs were observed [2–4]. In approximately 25 %, IVH remains asymptomatic and can only be discovered on imaging procedures. Significant advances in technology of cranial ultrasonography (universality, availability, no risk of radiation exposure, and non-invasive procedure) allow it to be a good choice for early identification of significant intracranial lesions in healthy full-term neonates [2–4].

### Etiology and risk factors for IVH in full-term neonates

Most IVHs arise from the posterior tufts at the glomus in the choroid plexus, and less commonly arise from the small residual germinal matrix tissue near the thalamocaudate groove, the thalamus, and the watershed area of the foramen of Monro near the caudate nucleus. The bleedings with higher grades originated more often from choroid plexus [3, 5]. Some arteriovenous malformations (AVM) may cause neonatal intracranial hemorrhage. Reports of AVM of choroid plexus have been limited to descriptions of individual cases [6]. IVH may be followed by development of venous sinus, medullary, or cortical vein thrombosis [7]. In one third of cases, a hemorrhage mechanism cannot be determined from neuroimaging studies [5]. Multifocal intraparenchymal hemorrhage was found in the first reported case and choroid plexus hemorrhage in the second patient.

IVH bleeding in the full-term newborn may occur as a result of birth asphyxia and resuscitation at birth [3, 5, 8, 9]. A significant clinical problem, which is generally regarded as one of the risk factors of IVH, is asphyxia, a secondary consequence to an interruption of placental blood flow and decreased blood flow to the brain and impairment of cerebral autoregulation. Treatment with therapeutic hypothermia in neonates with hypoxic ischemic encephalopathy may prevent the development of severe brain injuries and improve the long-term outcome. On the other hand, it places newborns at greater risk of IVH by causing fluctuations of the cerebral blood flow, depressed cardiac function, hypotension, and changes in coagulation cascade [3]. It was demonstrated that severe stages of HIE may lead to development of severe IVH [10]. Fetal distress was identified as a significant risk factor for IVH and may compromise the vascular environment and may be the first sign of fetal compromise due to IVH in utero [5, 11]. However, since IVH itself may cause respiratory distress, it is difficult to demonstrate that asphyxia is a significant factor in IVH pathogenesis.

The vaginal birth process itself may be traumatic enough to cause IVH in term newborns. Moreover, instrumental deliveries have been shown as a risk factor for IVH in term newborns [12, 13], while other studies did not confirm this association [5].

Bleeding disorders are rare cause of IVH in term newborns. Thrombocytopenia (drug-induced, infectious, genetic, immune-related) is a condition that may lead to IVH [8]. Coagulation factors deficiencies (vitamin K-dependent coagulation factors deficiency, factor VIII and IX deficiency) have been implicated with intracranial hemorrhage [14, 15]. Diagnosing and treating coagulopathic changes may help to decrease the risk for IVH. Coagulopathy significantly increases the risk for IVH, especially in patients treated with extracorporeal membrane oxygenation, therapeutic hypothermia, and in children with severe congenital heart diseases (secondary to altered hemodynamics or anticoagulant administration) [5].

Newborns described in our study were delivered vaginally in good clinical condition, without any symptoms of asphyxia. Congenital heart diseases, infections, and coagulation disorders were also excluded. Characteristic of patients, clinical presentation, risk factors, and source of bleeding in term newborns previously reported with IVH are shown in Table 1.

**Table 1:** Characteristic of patients, clinical presentation, risk factors, and source of bleeding in term newborns previously reported with IVH

	Number of patients	Median gestational age	Median day at presentation	Clinical presentation	Risk factors	Source of bleeding
Bruno et al. (2014) [5]	42: IVH (10 %), IPH (26 %), IVH + IPH (64 %)	39.7 weeks	1 day	Seizure (64 %) Apnea (29 %) Poor oral intake (29 %) Fever (14 %)	Congenital heart disease (26 %) Fetal distress (25 %) ECMO (10 %) Sepsis (2 %) None identified (60 %)	Choroid plexus (19 %) Germinal matrix (19 %) Hemorrhagic transformation of arterial ischemic stroke (24 %) or venous infarction (29 %) or arterial watershed injury (5 %) Unknown (29 %)
Afsharkes et al. (2015) [4]	30: IVH (100 %)	38.7 weeks	3.9 days	Seizure (50 %) Poor oral intake (30 %) Fever (10 %)	Coagulation disorders (50 %)	Choroid plexus (60 %) Germinal matrix (20 %) Parenchyma (67 %) Unknown (13 %)
Ou- Yang et al. (2010) [17]	24 ICH: IVH/IPH (50 %)	NA	2 h to 11 days	Seizure (46 %) Cyanosis (29 %) Tachypnea (21 %) Poor oral intake (4 %) Fever (4 %)	NA	NA

*ICH* intracranial hemorrhage, *IVH* intraventricular hemorrhage, *IPH* intraparenchymal hemorrhage, *ECMO* extracorporeal membrane oxygenation, *NA* not available

Today, the attention paid to genetic factors is increasing in the development of IVH but their connection with IVH in the newborn has been confirmed by few clinical studies, especially in preterm newborns. Polymorphisms in the Factor V Leiden gene were associated with the atypical timing of IVH. The methylenetetrahydrofolate reductase (MTHFR) variant 677C > T polymorphism and a low 5-min Apgar score additively increased the risk of IVH in preterm neonates [16]. It should be noted that none of the patients in our study present with factor V Leiden 1601G > A, MTHFR 677C > T; 1298A > C polymorphisms.

### Outcomes following IVH in full-term neonates

Neurodevelopmental impairment has been reported in nearly 50 % of patients with IVH. More than half of survivors required physical therapy, and nearly half of them demonstrated neurologic deficits. However, more than half of them present with only mild neurological problems [5, 12, 17].

### Treatment options for IVH

The treatment of newborns with IVH should be done by a multidisciplinary team consisting of pediatricians, neuroradiologists, pediatric neurologists, and neurosurgeons. Treatment should be focused on adequate ventilation, feeding, prevention of metabolic acidosis, and normalization of coagulation disorders. Anticonvulsant therapy should be used to control seizure activity. Transfusion with fresh frozen plasma and platelets may be beneficial in newborns with IVH. Neurosurgical intervention in patients with IVH should be considered in posthemorrhagic hydrocephalus to relieve increasing intracranial pressure or to drain hemorrhagic ventricular cerebrospinal fluid [18].

In conclusion, several factors influence the predisposition for severe IVH in term neonates. A perinatal period complicated by fetal distress, birth trauma, and severe asphyxia should be taken into account. However, it is possible that etiopathogenesis cannot be defined clearly as in our cases. Cranial ultrasounds in a specific group of term newborns (taking into account risk factors for IVH) should be widely recommended.
